# SynerClust: a highly scalable, synteny-aware orthologue clustering tool

**DOI:** 10.1099/mgen.0.000231

**Published:** 2018-11-12

**Authors:** Christophe H. Georgescu, Abigail L. Manson, Alexander D. Griggs, Christopher A. Desjardins, Alejandro Pironti, Ilan Wapinski, Thomas Abeel, Brian J. Haas, Ashlee M. Earl

**Affiliations:** ^1^​ Broad Institute, Cambridge, MA, USA; ^2^​ enEvolv, Boston, MA, USA; ^3^​ Delft University of Technology, Delft, The Netherlands

**Keywords:** orthogroup clustering, orthologues, comparative genomics, synteny

## Abstract

Accurate orthologue identification is a vital component of bacterial comparative genomic studies, but many popular sequence-similarity-based approaches do not scale well to the large numbers of genomes that are now generated routinely. Furthermore, most approaches do not take gene synteny into account, which is useful information for disentangling paralogues. Here, we present SynerClust, a user-friendly synteny-aware tool based on synergy that can process thousands of genomes. SynerClust was designed to analyse genomes with high levels of local synteny, particularly prokaryotes, which have operon structure. SynerClust’s run-time is optimized by selecting cluster representatives at each node in the phylogeny; thus, avoiding the need for exhaustive pairwise similarity searches. In benchmarking against Roary, Hieranoid2, PanX and Reciprocal Best Hit, SynerClust was able to more completely identify sets of core genes for datasets that included diverse strains, while using substantially less memory, and with scalability comparable to the fastest tools. Due to its scalability, ease of installation and use, and suitability for a variety of computing environments, orthogroup clustering using SynerClust will enable many large-scale prokaryotic comparative genomics efforts.

## Data Summary

Genome assemblies for the *Escherichia coli* dataset were downloaded from GenBank (Table S1, available with the online version of this article). Genome assemblies for the *Mycobacterium tuberculosis* and *Enterobacteriaceae* datasets were sequenced at the Broad Institute and have been submitted to GenBank (Table S1). The SynerClust tool is available at https://synerclust.github.io and a Docker image is available at https://hub.docker.com/r/synerclust/synerclust/.

**Impact Statement**

While large genomic studies promise to unlock critical insights into the biology and evolution of microbes including, for example, antibiotic-resistant bacteria, they require that computational tools also scale to process large volumes of data quickly, accurately and at reasonable computational expense. Orthology prediction underpins many comparative genomics studies, and is important for classifying and assigning functions to genes, since this reveals important aspects of gene biology and evolution. Current orthologue prediction tools struggle to quickly and accurately predict orthologues from large collections of microbial genomes. SynerClust is a new, easy-to-use tool that enables more complete and rapid identification of orthologues and paralogues in large datasets of thousands of bacterial genomes. Their accurate identification enables reconstruction of more reliable phylogenetic trees, inference of gains and losses of specific genes over evolutionary time, and identification of sets of core genes that define a group of organisms, such as a species.

## Introduction

The number of sequenced microbial genomes has grown exponentially. Comparative genomic datasets now routinely include thousands of genomes, drastically increasing or rendering prohibitive the compute time and memory usage for popular orthologue clustering tools. In particular, tools that rely upon all-vs-all blast searches, including rbh, based on reciprocal best blast hits [1], as well as OrthoMCL [2], PanOCT [3] and pgap [4], have compute times that scale at least quadratically with input and may require CPU (central processing unit) weeks or years for large datasets [5], and also require prohibitively large amounts of memory. Currently, the most scalable orthologue reconstruction algorithms are: Hieranoid2 [6], which uses a species guide tree with a stepwise approach; Roary [7], which uses cd-hit [8] to pre-cluster sequences; ls-bsr [9], which uses tblastn or blastn; and PanX [10], which uses Diamond [11] and subdivides the dataset to perform alignments.

Most existing scalable tools, including Hieranoid2, ls-bsr and PanX, do not make use of synteny, i.e. conserved gene order. Particularly valuable for bacterial genomes with operon structure [12] and high gene density [13], synteny can help to discriminate between paralogues to improve the accuracy of orthologue clusters (or orthogroups) [14, 15]. Several existing tools use synteny [7, 16, 17], including Roary and synergy [18], which has been applied to yeast [19] and *Mycobacterium* [20]. However, with the exception of Roary, which was developed for use on closely-related genomes [7, 10], tools that incorporate synteny were not designed to scale to large datasets. With the goal of designing a scalable algorithm capable of accurately clustering a wider range of genomes quickly, we adapted the original synergy algorithm into a new, open-source orthologue clustering tool called SynerClust, integrating features to deal with challenges encountered in bacteria, such as horizontal gene transfer. In benchmarking, SynerClust was able to rapidly and more completely identify sets of core genes for datasets that included diverse strains, and used substantially less memory than other tools.

## Methods

### Algorithm

The original synergy algorithm uses a combination of sequence similarity, synteny and parsimony to reconstruct the most likely orthogroups and their phylogenies at each node of a guide tree [18]. Starting from the results of an all-vs-all blast search, the algorithm reconstructs orthogroups for each common ancestor from tip to root by scoring all possible trees based on the implicit number of gain and loss events and the conservation of synteny and homology. However, synergy was not scalable or made available as an easily accessible open source software. Here, we implement SynerClust to further build on the success of synergy, as well as to enable scalable execution and make the software easily accessible.

**Fig. 1.**
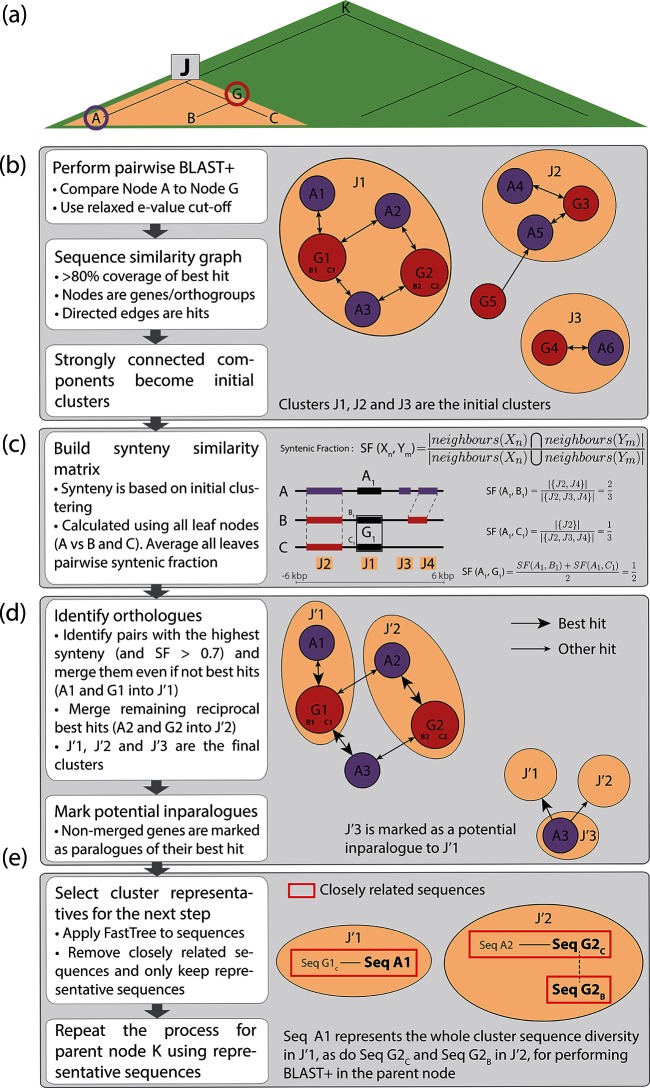
Overview of the SynerClust algorithm. (a) Input phylogeny: example of a phylogenetic guide tree. SynerClust traverses the input phylogeny from the leaves to the root, iteratively computing sequence similarity and synteny, combining information from the children of each internal node. First, leaves B and C (children) are processed at internal node G (parent). Second, node G and leaf A are processed at internal node J. This second step is used as an example in the algorithm explanation below. (b) Initial clustering for node J: initial clusters of orthogroups are constructed from blast+ results between representative sequences of child orthogroups. A lenient cut-off (*E* value 1×10^−5^) is used, and hits with at least 80 % identity to the best hit are kept. After filtering, only reciprocal hits are used to build a graph from which each set of connected orthogroups becomes a cluster (orange groups). (c) Calculation of syntenic fraction: a syntenic fraction for a specific orthogroup (orthogroup coloured in black) is calculated by dividing the number of shared neighbours within a 6 kb distance window (coloured in purple or red) by the total number of neighbours between two genomes (shared or unshared). For each cluster, a syntenic similarity matrix is built using the mean of all pairwise syntenic fractions. (d) Final clustering: final orthogroups for the current parent node are defined from the initial clusters by first looking for highly syntenic pairs, then for remaining pairs of reciprocal best hits. Child orthogroups that remain unmerged are marked as paralogues (potential inparalogues) of their best hit. At the next node, if they are still not part of an orthogroup, the mark is kept; otherwise it is removed. (e) Representative selection: for each parent orthogroup, representative sequences from child orthogroups are aligned (using muscle [35]) and used to build a tree (using FastTree2 [21]). Groups of highly similar sequences are defined by applying a sequence similarity threshold (red boxes). The longest sequence is then selected as a representative for all other sequences within a set mutational distance. This is repeated by selecting additional representatives until all sequences are represented.

To increase scalability, synergy was modified to select representative sequences for each orthogroup at every internal node of the guide tree (Figs 1a–e, and S1). Using only a subset of sequences decreases the search space and, thus, run-time. Once orthogroups are identified for the children of a particular node (Fig. 1e), FastTree2 [21] is used to compute a phylogenetic tree of all sequences within each orthogroup at that node, and a distance threshold is used to determine how many representative sequences are used in subsequent steps.

To improve the accuracy of orthologue and paralogue classification, SynerClust first groups together the most syntenic orthogroup pairs, then adds the remaining most similar pairs to build final clusters. SynerClust additionally delays merging paralogues until the algorithm reaches the root node, so that inparalogues, defined as genes that arose from a duplication that occurred after the most recent common ancestor (MRCA), can be distinguished from outparalogues, defined as genes that arose from a duplication that predated the MRCA. These modifications prevent the generation of clusters that are too large due to the inclusion of improperly classified inparalogues (see the Supplementary Material).

### Benchmarking

In order to compare the quality of orthogroups obtained using SynerClust to those obtained using other tools, we examined consistency of gene functional annotations within orthogroups for the *Escherichia coli* and *Enterobacteriaceae* datasets using previously established orthology benchmarking metrics (http://orthology.benchmarkservice.org/cgi-bin/gateway.pl) [22], which included the mean Schlicker similarity score [23] for gene ontology (GO) terms [24] and Enzyme Commission (EC) [25] numbers (see the Supplementary Material). As GO and EC annotations were available for <50 % of clusters, we also calculated analogous functional similarity metrics based on kegg (Kyoto Encyclopedia of Genes and Genomes) [26] and Pfam [27] annotations, which were available for >75 % of clusters (see the Supplementary Material).

## Results

**Fig. 2.**
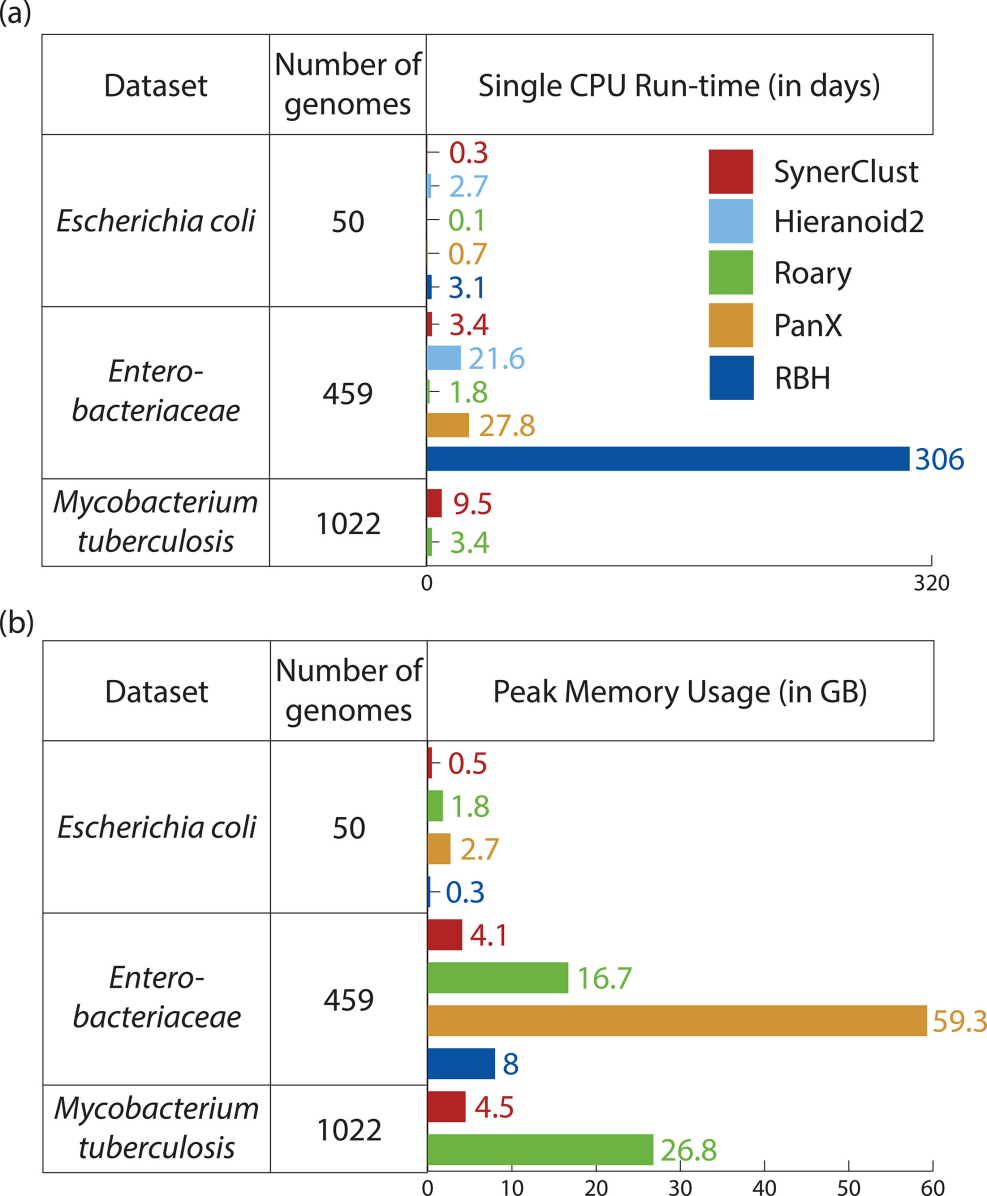
SynerClust runs fast and uses less memory than other tools. (a) Run-times indicate estimated CPU time (for details see Table S2). (b) Memory usage value indicated is the peak value.

To assess SynerClust’s speed and scalability, we compared its run-time and clustering quality to those of four orthologue clustering tools selected to represent popular or scalable algorithms: rbh [1], Hieranoid2 [28], Roary [7] and PanX [10]. When possible, all tools were run on three test datasets representing organisms having different genome sizes, sequence divergence and syntenic conservation (Fig. 2, Table S1): a small set of highly curated *E. coli*; a larger, more diverse set of *Enterobacteriaceae* covering five genera; and a dataset of over 1000 highly syntenic *Mycobacterium tuberculosis* strains.

In our benchmarking, SynerClust and Roary were by far the fastest and most scalable tools in terms of CPU time (Fig. 2a), and run-time for both tools scaled similarly with dataset size. However, Roary used substantially more memory than SynerClust (Fig. 2b), and Roary’s memory usage did not scale as well with dataset size. SynerClust’s run-time and memory usage both were highly scalable, since its overall run-time grew linearly with the proportion of unique genes per genome (Table S2). The run-times of rbh, Hieranoid2 and PanX increased drastically on our *Enterobacteriaceae* dataset, especially for rbh (Fig. 2a), as did the memory usage for PanX (Fig. 2b), showing substantially less capacity for scaling (see the Supplementary Material). Therefore, we did not run rbh, Hieranoid2 or PanX on our largest dataset, because their requirements exceeded reasonable CPU or memory availability.

**Fig. 3.**
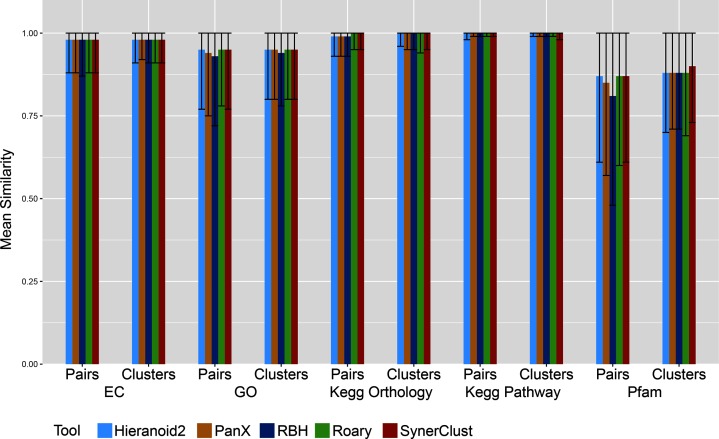
Consistency of function within SynerClust orthogroups is similar to that of other methods. Scoring metrics for different tools on the *E. coli* dataset: mean Schlicker EC score, mean Schlicker GO score, kegg orthology Jaccard similarity, kegg pathway Jaccard similarity and Pfam Jaccard similarity. ‘Pairs’ indicates that a mean is taken over all pairwise combinations, whereas ‘clusters’ indicates a mean over the clusters. Error bars represent the sd. Similar results are seen for the *Enterobacteriaceae* dataset (Fig. S3).

**Fig. 4.**
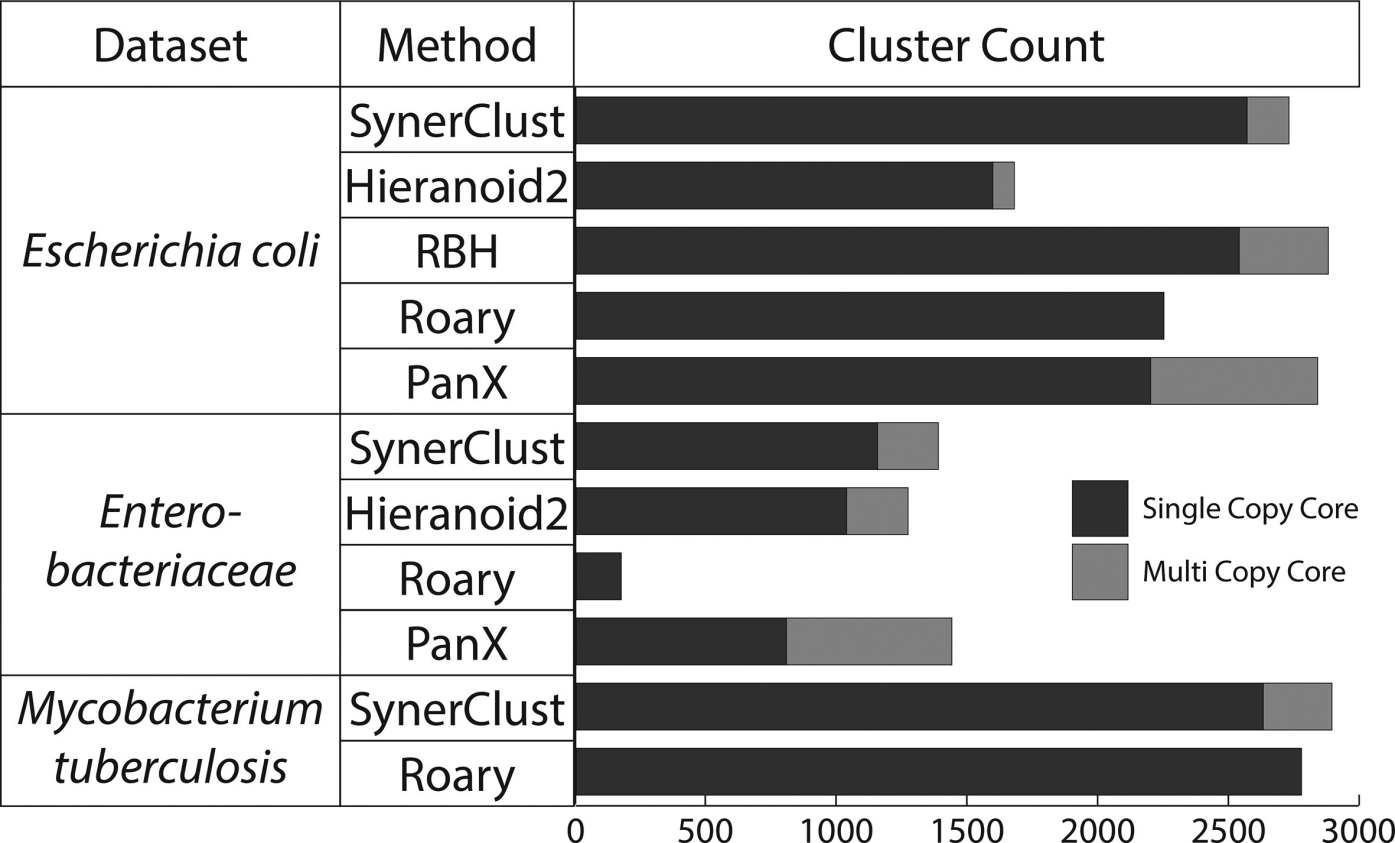
SynerClust consistently identifies a large SCC across all datasets. The numbers of SCC and multi copy core orthogroups identified by each method are shown. We did not run Hieranoid2, PanX or rbh on the *M. tuberculosis* dataset because these methods do not scale well enough to run on datasets of this size.

Based on our functional annotation metrics (see Methods), all tested tools performed similarly, indicating that they all worked well in grouping genes having similar functional annotations (Figs 3, S2 and S3). However, the number and size of orthologue clusters varied among tools. Importantly, many comparative genomics analyses are dependent upon accurate calculation of a single copy core (SCC) to highlight core conserved functions among groups of organisms and to serve as a substrate for phylogenetic analysis. SynerClust consistently yielded one of the largest SCCs for each of our benchmarking datasets (Fig. 4). For the more diverse *Enterobacteriaceae* dataset, SynerClust produced the largest number of SCC clusters, whereas Roary significantly under-clustered orthologues (see the Supplementary Material), resulting in an unrealistically low set of 172 SCC genes as compared to SynerClust’s 1156 genes (Fig. 4). This is consistent with previous observations that Roary works best when clustering closely-related genomes [10]. For the more closely-related, single-species *E. coli* dataset, SynerClust yielded a 14 % larger SCC than Roary, while Hieranoid2 substantially under-clustered orthologues (Table S3), resulting in substantially (38 %) fewer SCC clusters than SynerClust (Figs 3 and S4, Table S3, Supplementary Material). Finally, on the largest and most closely-related *M. tuberculosis* dataset, Roary generated a slightly (6 %) larger SCC than SynerClust, but used far more memory (600 % more; Fig. 2b). Of the 291 SCC genes unique to Roary, 80 % had large size discrepancies (>50 % of the longest gene length), including examples of orthogroups containing sequences that measured as little as 10–20 % of the length of the others in the same cluster; the majority of the rest belonged to *M. tuberculosis* repetitive gene families known to be difficult to sequence and analyse. Of the 144 SCC genes unique to SynerClust, only 5 % had large size discrepancies (>50 % of the gene length) and 3 % represented repetitive gene families.

## Discussion

Our benchmarking results showed that SynerClust is able to rapidly identify orthologue relationships in bacteria at least as completely as previous tools, using a fraction of the memory (Table S2), on all three of our test datasets. Only Roary was faster; however, this came at a cost of higher memory usage and lower clustering quality, particularly when applied to a more diverse strain set. SynerClust was built on a version of synergy with improved scalability, Synergy2 (http://synergytwo.sourceforge.net), that introduced representative sequences and has been applied to studies of *Fusobacterium* [29] and the enterococci [30]. However, for gene families with multiple paralogues, Synergy2 could not readily distinguish inparalogues from outparalogues, and was not sufficiently scalable. This motivated the development of SynerClust, with additional improvements that made it amenable to orthogroup clustering of thousands of genomes.

SynerClust makes efficient use of computational resources by replacing all-vs-all sequence-similarity computations with representative subsets. Incorporating synteny for disentangling paralogues increases accuracy by giving the orthogroup clustering algorithm increased ability to pinpoint the correct orthologue from among a set of paralogues, which helps to compensate for inaccuracies that may result from using representative sequences, which is essential for scaling (see the Supplementary Material).

SynerClust is made more robust to errors in the input phylogeny and to gene loss events by delaying merging of paralogues until after all available information has been taken into account in the last algorithm step, allowing for more accurate orthogroup refinement. As SynerClust traverses the tree from leaves to root, genes that appear to be inparalogues at early steps find appropriate orthologues at later steps as the algorithm approaches the root (Fig. S5). This situation can occur when one branch of the phylogeny has lost a paralogue, or when a strain contains genes obtained through horizontal gene transfer.

The ability to rapidly obtain a more complete SCC is critically important for comparative genomics and accurate reconstruction of phylogenies. In both our *E. coli* and *Enterobacteriaceae* datasets, SynerClust identified the largest set of SCC clusters without sacrificing cluster size, while maintaining fast run-time. In contrast, Roary’s performance on the diverse *Enterobacteriaceae* dataset was greatly reduced, and both Hieranoid2 and PanX had substantially longer run-times. We did not benchmark ls-bsr [9], as this tool has been shown to be less sensitive than Roary [7]. While further studies are needed to demonstrate that SynerClust’s high performance extends across all bacterial and eukaryotic datasets, we have shown that SynerClust has high performance across a wide range of dataset sizes and phylogenetic diversity.

For ease of use, we simplified installation and minimized software dependencies. On a typical Linux system, SynerClust only requires installation of blast+, Python 2.7, and the Python libraries Numpy and NetworkX. We also provide a Docker [31] image at https://hub.docker.com/r/synerclust/synerclust/. The user will normally not need to alter default settings and the software can be run in series or parallel. Alternate sequence similarity search tools such as Blat [32], Diamond [11], cd-hit [8] or ublast [33] could be used instead of blast+, potentially allowing for even faster run-times. While a guide phylogenetic tree is needed as input to determine the order in which nodes are compared, we sought to make SynerClust user friendly and able to work from different starting points in terms of knowledge of the dataset phylogeny: if an accurate tree is unavailable, SynerClust can be run iteratively, first using an approximate tree (generated using amphora marker genes [34] or using a k-mer based approach), and then using a tree built from the SCC clusters generated (see the Supplementary Material). In addition, it is simple to expand an initial dataset and only perform computation for the newly added species or groups of species. SynerClust is freely available at https://synerclust.github.io.

## Data bibliography

1. Source code for SynerClust is available on GitHub; https://synerclust.github.io/
2. A SynerClust Docker image is available on Docker Hub; https://hub.docker.com/r/synerclust/synerclust/
3. A full listing of NCBI accessions for strains used in this paper is available in Table S1.

## Supplementary Data

Supplementary File 1

Supplementary File 2
