# Supplementary material for: SynerClust: a highly scalable, synteny-aware orthologue clustering tool
Source: Microb Genom. 2018 Nov 12;4(11):e000231. doi: 10.1099/mgen.0.000231 (PMC6321874; doi:10.1099/mgen.0.000231)
Supplement: Supplementary File 2 [file mgen-4-231-s002.pdf]

Supplementary Methods and Details of SynerClust Algorithm

SynerClust: a highly scalable, synteny-aware ortholog clustering tool

Christophe H. Georgescu[1], Abigail L. Manson[1], Alexander D. Griggs[1], Christopher A. Desjardins[1], Alejandro Pironti[1], Ilan Wapinski[3], Thomas Abeel[1,2], Brian J. Haas[1], Ashlee M. Earl[1].

[1] The Broad Institute of MIT and Harvard, Cambridge, Massachusetts, USA.
[2] Delft University of Technology, Delft, The Netherlands.
[3] enEvolv, Boston, Massachusetts, USA.

Corresponding Authors:
Dr. Ashlee M. Earl
The Broad Institute of MIT & Harvard
415 Main Street
Cambridge, MA 02142, USA
E-mail: aearl@broadinstitute.org
Phone: +1 (617) 714-7927

Brian J. Haas
The Broad Institute of MIT & Harvard
415 Main Street
Cambridge, MA 02142, USA
E-mail: bhaas@broadinstitute.org
Phone: +1 (617) 714-8532

Supplementary Methods

Genome Annotation

In order to assure consistency among the genome sequences used for benchmarking, all genomes were re-annotated in a uniform manner using the Broad Institute's prokaryotic annotation pipeline [1]. The protein-coding genes were predicted with Prodigal [2] and filtered to remove genes with >=70% overlap to tRNAs or rRNAs. The gene product names were assigned based on top blast hits against SwissProt protein database (>=70% identity and >=70% query coverage). Additional annotation analyses performed include PFAM [3], KEGG [4], GO [4], EC [5].

Construction of Guide Phylogenetic Trees

Initial phylogenetic trees were constructed for each dataset to use as input to the clustering algorithms. For the *Enterobacteriaceae* dataset, a core gene phylogeny was constructed using FastTree2 [6], based on single-copy core genes obtained from RBH [7] and aligned with MUSCLE [8]. Kmer-based trees were constructed for the *E. coli* and *M. tuberculosis* datasets. A distance matrix was built based on Jaccard similarities of all 15-mers with a kmer tree tool [9], then a neighbour-joining tree was built with the R library phytools [10].

Estimation of Memory Requirements for RBH

For the *E.coli* dataset (50 genomes) containing 245,217 genes, RBH required 339MB of random-access memory (RAM) to run. For the *Enterobacteriaceae* dataset (459 genomes) containing 2,310,882 genes, RBH required 8.4GB of RAM to run. Considering that the *M. tuberculosis* dataset (1,022 genomes) contained 6,318,055 genes and that memory requirements scale quadratically with the number of genes, we estimated that RBH would require 60GB of RAM in order to run on this dataset.

Roary performance on the *Enterobacteriaceae* dataset

The Roary documentation suggests the lowest value that should be used for the percent identity parameter is 90%. When trying values between 90% and 95% for the *Enterobacteriaceae* dataset, the highest SCC that we obtained was only 172 clusters for a 90% identity value, compared to over a 800 for all other methods. In order to achieve a more in-depth benchmarking, we also compared results when using an identity threshold of 50%, despite strong warnings against doing so in the Roary documentation *e.g.*, "not advised to go below 90% unless you know what you're doing" and "if you are setting this below 70%, then you're doing something wrong." Although we have not included these results in our main tables, we observed

that the SCC size increased to 1,062, which was much closer to that found using other methods. However, when assessing these clusters using our scoring metrics, we observed overall lower cluster quality, especially for the Pfam per cluster score (Fig. S3).

Details of the SynerClust algorithm

Pseudo-code

```
node = root
solve(node)

solve(node) {
    if node is a leaf:
            return
    for child in children:
            solve(child)
            if child is a leaf:
                    self_blastp(child)
    blastp(child[0], child[1])
    blastp(child[1], child[0])

    filter_blastp_results()
    remove_non_reciprocal_blastp_results()
    build_graph_from_blastp_results()

    initial_clusters = select_connected_orthogroups_in_graph()
    for cluster in initial_clusters:
            build_synteny_distance_matrix()
            final_clusters.add(cluster_most_syntenic_pairs())
            final_clusters.add(cluster_remaining_reciprocal_best_hits())
            final_clusters.add(single_orthogroups)
            mark_single_orthogroups_as_potential_inparalogues()

    for cluster in final_clusters:
            select_representative_sequences()

}
```

1. Preparation for Clustering

A full list of all available options is detailed in the README on the GitHub (https://github.com/SynerClust/SynerClust). Every parameter that has a default value can be changed by the user.

Generation of a directory structure. The first preprocessing step is to generate a folder identifier for each leaf and node of the input species phylogenetic tree. For leaves, the name is a hash of its genome name. For internal nodes on the phylogeny, the name is a hash of the combination of the names of both of its children. Structuring the naming system in this manner means that SynerClust can be run multiple times using different input phylogenetic trees, without having to reprocess unchanged portions of the phylogeny. As long as the name of the genome (for leaves) or of the children (for internal nodes) remains the same, the identifier for this node will also be the same, even if other portions of the phylogeny have been altered. This structure is important because it allows the user to easily modify a small portion of the phylogeny, or add a single genome, without redoing the entire calculation (Fig. S1). A useful application of this feature is that a quick and approximate phylogeny can be used as input for an initial clustering to determine single copy core genes. This single copy core can then be used to calculate a more accurate phylogeny, and a second run of SynerClust can be performed to refine the orthogroup clustering, in which portions of the tree that have not changed do not need to be recomputed.

Synteny calculations. The second preprocessing step is to read the sequence and annotation files for each input genome to extract all peptide sequences and generate synteny data. Using the syntenic window value defined by the user (default value 6,000 bp), SynerClust looks both upstream and downstream of each gene to identify other genes that have their center within the syntenic window. All such neighbouring genes are identified as syntenic to the current one. Synteny information for each gene is written to a file.

2. Initial Clustering for Each Node

Once the preprocessing steps are complete, SynerClust can begin computing orthogroups at each node whose child nodes or leaves' orthogroups have been solved. Therefore, the next steps apply at each node individually (Figure 1).

All-vs-all BLASTP. The first step in the orthogroup calculations is to perform an all-vs-all BLASTP search between all protein sequences from the two children of the current node, which is equivalent to two pairwise BLASTP between two genomes. For children that are also leaves, a self-BLASTP is also performed in order to identify paralogues. At this step, the E-value threshold used is very lenient (default 1e-5; at least 50% identity over 50% length of target) in order to be inclusive of potentially important hits that could be missed due to the use of representative sequences – a feature of SynerClust that reduces the search space and allows scalability.

Constructing the graph from BLASTP [11] results. To model the BLASTP results, all orthogroups from child nodes are used to build a graph, and all reciprocal best hits are added as directed edges from the query node to the target node. Additional hits are filtered to keep only those that are reciprocal and whose adjusted percent identity relative to the best hit are above a defined threshold (default 80%). The adjustment to the percent identity is based on the size of the longest sequence (query or target). This step is critical to avoid using hits from sequences that are too far apart relative to others, or that are between sequences with lengths that vary too much. The hits that remain are also added as directed edges to the graph. All edges carry both the information of their rank and adjusted percent identity.

Construction of initial clusters. From this graph, SynerClust takes strongly connected components, defined as portions of the directed graph where there is a path from any node to any other node, using Tarjan's strongly connected components algorithm [12]. These subgraphs will represent the initial clusters of homologous and paralogous orthogroups, which will be refined in subsequent steps in order to distinguish the types of relationships between them. In addition, they will allow SynerClust to estimate synteny during the refinement step.

3. Final Cluster Construction for Each Node

The previous step allowed SynerClust to identify roughly as many clusters as there are closely related gene families. For the final cluster construction, or refinement step, each cluster is processed independently. In SYNERGY, gene trees are reconstructed using a scoring function which takes into account sequence similarity, gene synteny, and gene gains and losses. However, for this to work well, *a priori* knowledge of how the group of organisms being studied has evolved is recommended to adjust weights for accuracy. For example, at species branchings where there is a known whole genome duplication, the gain probability should be increased, while the loss probability should be increased in subsequent branchings. Even then, genes that are part of large families tend to almost exclusively be classified as outparalogues, because the trees produced by Neighbour-Joining tend to be unbalanced at most levels due to non additivity of values. Larger trees also have a quickly rising computational cost to score all possible edges when placing the root. To address these issues, SynerClust proceeds as follows.

Computation of syntenic fraction. SynerClust computes the syntenic fraction (value between 0 and 1) of each pair of orthogroups present in the current initial cluster, in order to build a synteny distance matrix of the cluster. This is done by looking at the overlap of initial cluster assignments for all neighbouring genes with each member of the current initial cluster, using the synteny information computed during the pre-processing of each input genome. To prevent artificially high synteny due to similar genes that are neighbours (like transposases), we ignore neighbouring genes that are highly similar to the currently processed gene. The syntenic fraction formula is:

$$Syntenic\ Fraction\ (Ai, Gi) = \frac{|neighbours(Ai)\ neighbours(Gi)|}{|neighbours(Ai)\ neighbours(Gi)|}$$

where A_i and G_i are orthogroups solved from nodes A and G, respectively. Neighbours(A_i) and Neighbours(G_i) are multisets calculated as all genes within 6,000 bp upstream or 6,000 bp downstream of each gene composing the orthogroup A_i, or G_i, respectively, scaled by the number of leaves that compose A or G, respectively.

Cluster refinement. Cluster refinement is performed by combining information from the graph of reciprocal hits with the syntenic fractions. First, a list of highly syntenic pairs of genes (syntenic fraction > 0.7 by default) is produced. From this list, pairs in which both genes are only present in the list once are merged into a parent orthogroup node regardless of whether they are best hits are not. Parent orthogroup nodes keep all incoming edges from both merged child nodes, but discard all outgoing edges. For pairs where at least one of the genes is also part of another highly syntenic pair, a list of all these pairs is made and sorted by lower sum of BLASTP ranks and higher adjusted percent of identity (compared to the best hit). The first pair is then merged to a parent orthogroup.

After highly syntenic pairs of genes have been checked, the remaining child nodes are merged to their reciprocal best hit to form orthogroups for the current node. If a member of a pair has multiple hits tied for best hit, SynerClust checks the difference in syntenic fraction between tied pairs and merges the pair with the highest syntenic fraction, if there is a noticeable difference (of at least 0.2 by default).

Resolving paralogues. At this stage, only ortholog relationships have been solved. In order to also solve paralogue relationships, the graph is checked for any remaining nodes that come from the children. However, unlike the commonly used method of merging paralogues that are closer to each other than to an ortholog, these nodes are added to a list of potential inparalogues with a reference to their best hit. The reason for this difference is that, although at a certain branching it may appear that orthogroups are inparalogues, they could actually be outparalogues, with a loss event having occurred in one of the children. If that is the case, when going up the phylogenetic tree of the compared species, the outparalogue lost in one of the children will be found again. However, as there is no spliting mechanism in ortholog clustering algorithms, there will be no way to correct for the incorrect clustering. For this reason, the lists of potential inparalogues from both children are then filtered to only keep those that have no ortholog in the current node. The main advantage of not merging paralogues and only marking them is to be able to evaluate whether they are inparalogues or outparalogues at the root, when having the most information, making SynerClust more robust to errors in the input phylogenetic tree and to loss events.

4. Selection of Representative Sequences

Now that the final orthogroups for the current node have been computed, the last step in the processing of a node is the key step that enables SynerClust's scalability: reducing the search space of the parent node by selecting representative sequences. To do this, SynerClust uses sequence distances based on the JTT substitution rates [13] computed by FastTree [6] to calculate the average distance of each sequence present in the orthogroup to the others. It then selects the longest sequence as a representative sequence for all other sequences in the orthogroup that are within a certain distance of this selected sequence (default mutation rate threshold is 1.2 from FastTree). This step is repeated until all sequences present in the orthogroup are represented, in order to preserve sequence diversity.

This allows the number of sequences that will be used for the BLASTP search at the parent node to increase, compared to one of the children, by only slightly more than the number of unique genes in the other children. For closely related genomes, the increase in number of sequences from leaves to root can be limited to just a few hundred. Even when analyzing very distantly related genomes, the sum of all BLASTP searches done can never exceed that of non-scalable clustering tools.

1. **Manson McGuire A, Cochrane K, Griggs AD, Haas BJ, Abeel T et al.** Evolution of Invasion in a Diverse Set of Fusobacterium Species. *mBio* 2014;5(6):e01864-01814.
2. **Hyatt D, Chen GL, Locascio PF, Land ML, Larimer FW et al.** Prodigal: prokaryotic gene recognition and translation initiation site identification. *BMC Bioinformatics* 2010;11:119.
3. **Finn RD, Tate J, Mistry J, Coggill PC, Sammut SJ et al.** The Pfam protein families database. *Nucleic Acids Res* 2008;36(Database issue):D281-288.
4. **Conesa A, Gotz S, Garcia-Gomez JM, Terol J, Talon M et al.** Blast2GO: a universal tool for annotation, visualization and analysis in functional genomics research. *Bioinformatics (Oxford, England)* 2005;21(18):3674-3676.
5. **Tian W, Arakaki AK, Skolnick J**. EFICAz: a comprehensive approach for accurate genome-scale enzyme function inference. *Nucleic Acids Res* 2004;32(21):6226-6239.
6. **Price MN, Dehal PS, Arkin AP**. FastTree 2 – Approximately Maximum-Likelihood Trees for Large Alignments. *PLOS ONE* 2010;5(3):e9490.
7. **Salichos L, Rokas A**. Evaluating ortholog prediction algorithms in a yeast model clade. *PLoS One* 2011;6(4):e18755.
8. **Edgar RC**. MUSCLE: multiple sequence alignment with high accuracy and high throughput. *Nucleic Acids Research* 2004;32(5):1792-1797.
9. Kmer tree. https://github.com/AbeelLab/kmertree [accessed.
10. Phytools. https://github.com/liamrevell/phytools [accessed.
11. **Camacho C, Coulouris G, Avagyan V, Ma N, Papadopoulos J et al.** BLAST+: architecture and applications. *BMC Bioinformatics* 2009;10:421.
12. **Tarjan R**. Depth-First Search and Linear Graph Algorithms. *SIAM Journal on Computing* 1972;1(2):146-160.
13. **Jones DT, Taylor WR, Thornton JM**. The rapid generation of mutation data matrices from protein sequences. *Computer applications in the biosciences : CABIOS* 1992;8(3):275-282.
14. **Zhang H, Gao S, Lercher MJ, Hu S, Chen W-H**. EvolView, an online tool for visualizing, annotating and managing phylogenetic trees. *Nucleic Acids Research* 2012;40(W1):W569-W572.

15. **He Z, Zhang H, Gao S, Lercher MJ, Chen W-H et al.** Evolview v2: an online visualization and management tool for customized and annotated phylogenetic trees. *Nucleic Acids Research* 2016;44(W1):W236-W241.

Figures

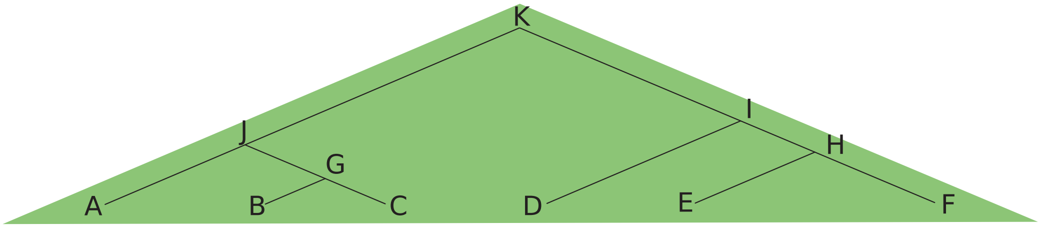

Fig. S1: **Example of an input phylogenetic tree used to guide the order in which SynerClust solves the orthogroups.** Leaves are the input extant species' genomes, while internal nodes are inferred extinct common ancestor genomes. In this case, SynerClust will first solve nodes G and H, then nodes J and I, and finally node K. This order is defined by the dependency of each node to its children--both child nodes must be solved before calculations can be begun on their parent node. Reciprocally, this also means that the processing of any independent nodes can be parallelized (i.e. nodes G and H). This also means that we can solve orthogroups for the two halves of the tree independently for different studies, and if we later decide to solve orthogroups for the whole tree, we only have one extinct node to compute. An example of the SynerClust algorithm applied to node J, whose two children are left node A and node G, can be found in Fig. 1 with the same coloring scheme.

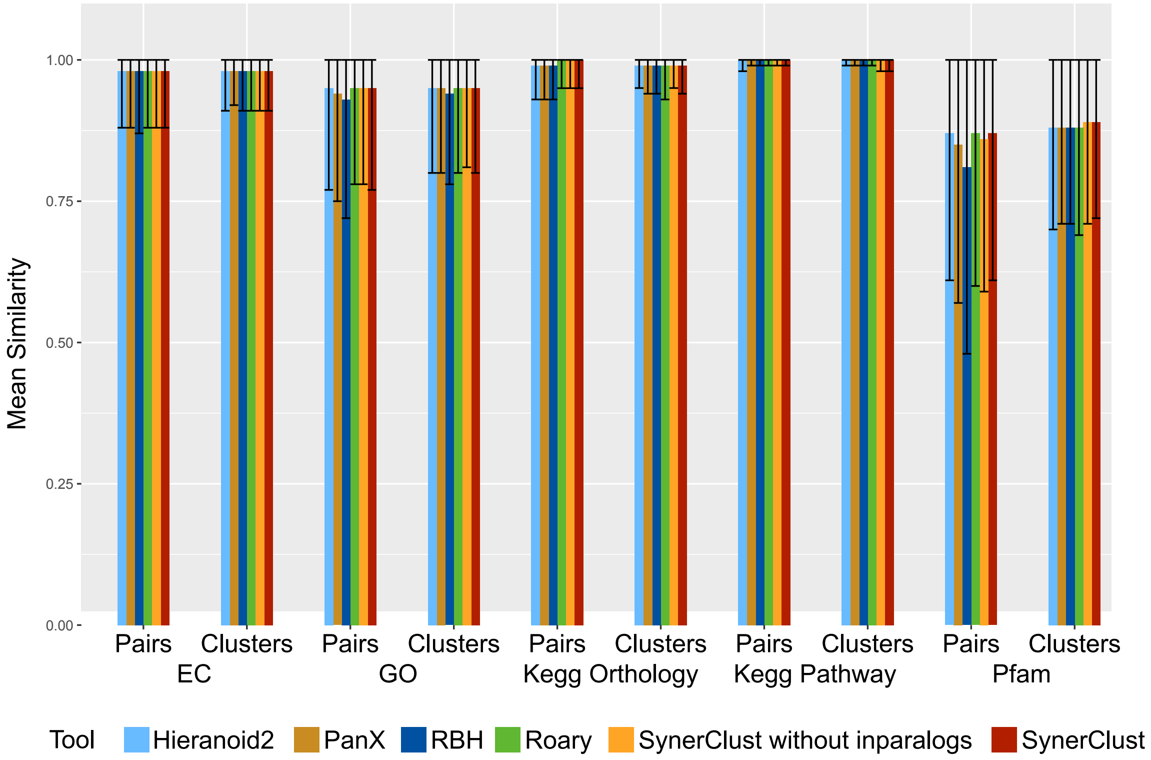

Fig. S2: **Consistency of function within SynerClust orthogroups is similar to that of other methods.** Scoring metrics for different tools on the *E. coli* dataset: Average Schlicker EC score, average Schlicker GO score, KEGG Orthology Jaccard similarity, KEGG Pathway Jaccard similarity, and Pfam Jaccard similarity. "Pairs" indicates that an average is taken over all pairwise combinations, whereas "Clusters" indicates an average over the clusters. Error bars represent the standard deviation. Similar results are seen for the Enterobacteriaceae dataset (Fig. S3)

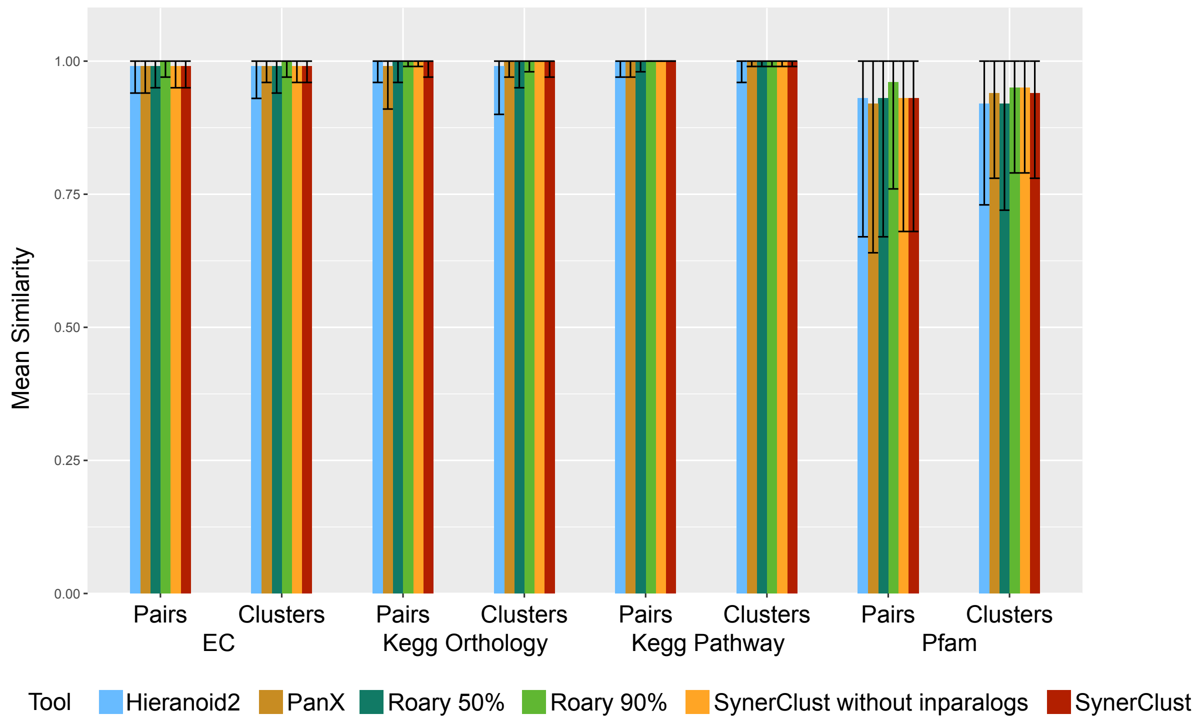

Fig. S3: **Consistency of function within SynerClust orthogroups is similar to that of other methods for the Enterobacteriaceae dataset. Scoring metrics for different tools:** Average Schlicker EC score, KEGG Orthology Jaccard similarity, KEGG Pathway Jaccard similarity, and Pfam Jaccard similarity. “Pairs” indicates that an average is taken over all pairwise combinations, whereas “Clusters” indicates an average over the clusters. SynerClust metrics are similar to those of other commonly used tools. Error bars represent the standard deviation.

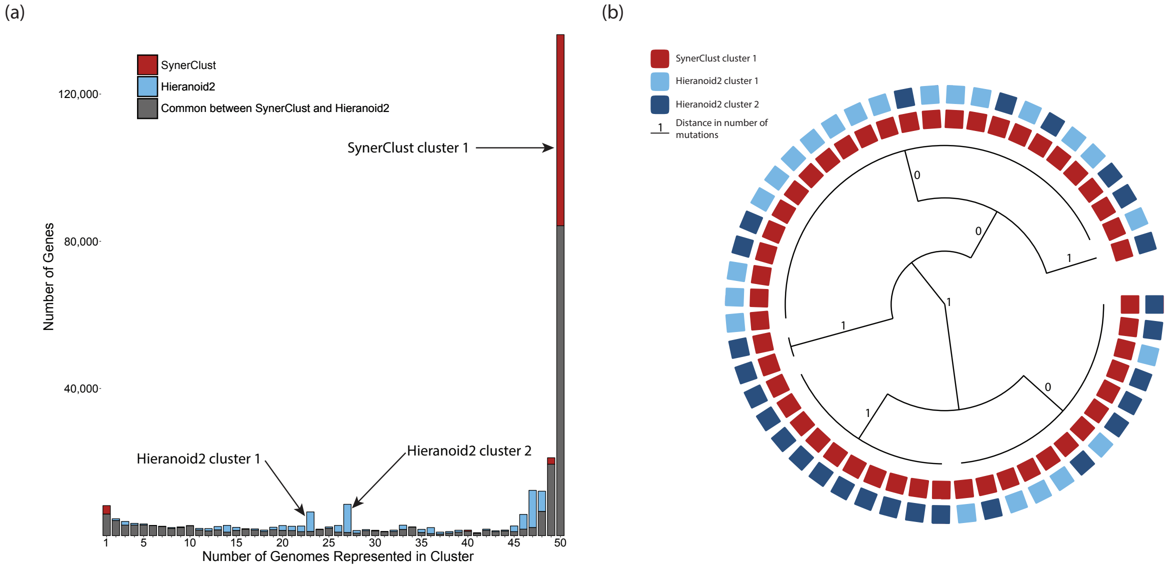

Fig. S4: **SynerClust provides a more complete view of the single copy core (SCC) than Hieranoid.** Of the Hieranoid2 SCC results, nearly all were identified by SynerClust; however, almost all (94%) of SCC orthogroups identified only by SynerClust were split into ≥2 clusters by Hieranoid2. Detailed inspection revealed that Hieranoid2 was not clustering closely-related genes as expected (Fig. S3), while SynerClust produced more SCC clusters that maximized both the sequence similarity of their members and their size (Table S3). (a) Total number of genes contained in clusters for SynerClust and Hieranoid2, as a function of the number of genomes represented in the cluster. The grey bars indicate gene counts common between the two methods, while the red and blue bars indicate additional gene counts for either SynercClust (red) or Hieranoid (blue). The largest difference is in the number of genes comprising core clusters (all 50 genomes are represented) that SynerClust correctly identifies (red), but Hieranoid2 splits over the full range of cluster sizes (blue). (b) An example of cluster identified as SCC by SynerClust (red) but kept in two clusters by Hieranoid2 (light and dark blue) is shown as a gene phylogeny [14] [15]. The numbers of amino-acid mutations are indicated in the branches of the tree. Note that Hieranoid2 assigns genes with identical sequences to different clusters while placing genes with different sequences in one cluster.

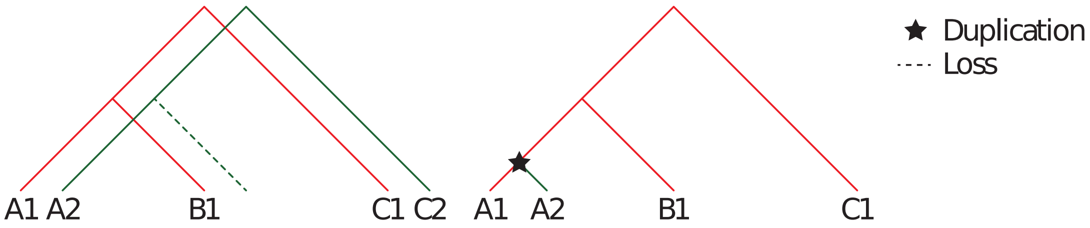

Fig S5: **Importance of delaying paralogue merging.** (a) Gene trees of two outparalogues (red and green). The dashed line represents a loss in organism B. If A1 and A2 are more similar to each other than either is to B1, they would be merged in a cluster as inparalogues when comparing A to B. (b) Gene trees of two inparalogues where the second gene arises from a duplication event (marked by a star) in a leaf organism. If the sequence similarity between A1 and A2 is lower than between A1 and B1, A2 will be considered an outparalogue and not clustered with A1.

Dataset			Single CPU runtimes (days)				
Name	Number of genomes	Number of genes	SynerClust	Hieranoid2	Roary	PanX	All-vs-All Blast
Escherichia coli	50	245217	0.3	3	0.1	0.7	3
Enterobacteriaceae	459	2310882	6.8 (realtime: 0.4 w/ 32CPUs)*	43.2 (realtime: 7.3 w/ 30CPUs)*	3.5 (realtime 0.6 w/ 8 CPUs)*	53.5 (realtime: 7.8 w/ 8 CPUs)*	306
Mycobacterium tuberculosis	1022	6318055	19*		6.8*		

Intel Xeon E5-2680 v2 @ 2.80GHz
* AMD Opteron(tm) Processor 6174 @ 2199.978MHz

Dataset			Maximal RAM usage (in GB)				
Name	Number of genomes	Number of genes	SynerClust	Hieranoid2	Roary	PanX	All-vs-All Blast
Escherichia coli	50	245217	0.5		1.8	2.7	0.3
Enterobacteriaceae	459	2310882	4.1		16.7	59.3	8
Mycobacterium tuberculosis	1022	6318055	4.5		26.8		

Table S2: (a) Table of the measured CPU times for each method and dataset (in days). Times were measured on an Intel Xeon E5-2680 v2 @ 2.80GHz (white background) and an AMD Opteron Processor 6174 @ 2199.978MHz (grey background). No runs of Hieranoid2, PanX or RBH were made on the *Mycobacterium tuberculosis* dataset because of compute time required or inability to parallelize certain steps. To account for the different CPU types used, the times on the AMD Opteron Processor 6174 @ 2199.978MHz were divided by two for Fig. 2a. This factor was based on the approximate PassMark (https://www.cpubenchmark.net/) CPU benchmarking score differences.

(b) Table of measured maximal Random Access Memory usage

All measures were made with the Linux tool "time", except for Hieranoid2 which provides its own benchmarking times but no memory usage.

	GO conservation test		GO conservation test per cluster		EC conservation test		EC conservation test per cluster	
	# ortholog relations	avg Schlicker	# ortholog relations	avg Schlicker	# ortholog pairs	avg Schlicker	# ortholog clusters	avg Schlicker
SynerClust without inparalogs	75,125	0.95+-0.17	4,493	0.95+-0.14	1,597,916	0.98+-0.10	2,566	0.98+-0.07
SynerClust	77,614	0.95+-0.18	4,562	0.95+-0.15	1,624,081	0.98+-0.10	2,400	0.98+-0.07
RBH	90,230	0.93+-0.21	4,407	0.94+-0.16	1,650,160	0.98+-0.11	2,367	0.98+-0.07
Hieranoid2	73,571	0.95+-0.18	4,934	0.95+-0.15	1,439,454	0.98+-0.10	3,209	0.98+-0.07
Roary	68,502	0.95+-0.17	4,783	0.95+-0.15	1,453,285	0.98+-0.10	3,065	0.98+-0.07
PanX	89,382	0.94+-0.19	4,460	0.95+-0.15	1,649,394	0.98+-0.10	2,343	0.98+-0.06

	Kegg Orthology		Kegg Orthology per cluster		Kegg Pathways		Kegg Pathways per cluster	
	# ortholog pairs	avg JS	# ortholog clusters	avg JS	# ortholog pairs	avg JS	# ortholog clusters	avg JS
SynerClust without inparalogs	424,061	1.00+-0.05	659	0.99+-0.04	424,061	1.00+-0.01	659	1.00+-0.02
SynerClust	427,611	1.00+-0.05	642	0.99+-0.05	427,611	1.00+-0.01	642	1.00+-0.02
RBH	430,533	0.99+-0.06	638	0.99+-0.05	430,533	1.00+-0.01	638	1.00+-0.01
Hieranoid2	379,836	0.99+-0.06	837	0.99+-0.04	379,836	1.00+-0.02	837	1.00+-0.01
Roary	384,855	1.00+-0.05	779	0.99+-0.06	384,855	1.00+-0.01	779	1.00+-0.01
PanX	432,379	0.99+-0.06	637	0.99+-0.05	432,379	1.00+-0.01	637	1.00+-0.01

	Pfam		Pfam per cluster					
	# ortholog pairs	avg JS	# ortholog clusters	avg JS	SCC	MCC	orphans	non-orphans clusters
SynerClust without inparalogs	4,413,865	0.86+-0.27	9,824	0.89+-0.18	2,702	0	8,515	12,508
SynerClust	4,701,749	0.87+-0.26	8,177	0.89+-0.17	2,574	161	5,300	10,633
RBH	5,616,870	0.81+-0.33	7,074	0.88+-0.17	2,544	342	5,338	9,042
Hieranoid2	4,359,645	0.87+-0.26	9,560	0.88+-0.18	1,598	83	5,880	12,122
Roary	3,799,348	0.87+-0.27	10,855	0.88+-0.19	2,255	0	8,890	11,576
PanX	5,101,638	0.85+-0.28	7,077	0.88+-0.17	2,204	641	4,141	6,303

Table S3: a) *Escherichia coli*

	EC conservation test		EC conservation test per cluster		Kegg Orthology		Kegg Orthology per cluster	
	# ortholog pairs	avg Schlicker	# ortholog clusters	avg Schlicker	# ortholog pairs	avg JS	# ortholog clusters	avg JS
SynerClust without inparalogs	118,125,838	0.99+-0.04	4,622	0.99+-0.03	39,025,250	1.00+-0.00	1,544	1.00+-0.02
SynerClust	120,957,943	0.99+-0.04	4,141	0.99+-0.03	40,099,743	1.00+-0.03	1,359	1.00+-0.03
Hieranoid2	122,556,949	0.99+-0.05	3,982	0.99+-0.06	40,391,510	1.00+-0.04	1,350	0.99+-0.09
Roary 90%	84,982,026	1.00+-0.03	9,993	1.00+-0.03	30,720,262	1.00+-0.01	2,993	1.00+-0.02
Roary 50%	111,014,211	0.99+-0.04	5,186	0.99+-0.05	36,550,488	1.00+-0.04	1,712	1.00+-0.05
PanX	126,924,892	0.99+-0.05	3,715	0.99+-0.03	43,018,578	0.99+-0.08	1,150	1.00+-0.03

	Kegg Pathways		Kegg Pathways per cluster		Pfam		Pfam per cluster	
	# ortholog pairs	avg JS	# ortholog clusters	avg JS	# ortholog pairs	avg JS	# ortholog clusters	avg JS
SynerClust without inparalogs	39,025,250	1.00+-0.00	1,544	1.00+-0.01	338,183,323	0.93+-0.25	27,394	0.95+-0.16
SynerClust	40,099,743	1.00+-0.00	1,359	1.00+-0.01	353,524,638	0.93+-0.25	21,681	0.94+-0.16
Hieranoid2	40,391,510	1.00+-0.03	1,350	1.00+-0.04	366,396,021	0.93+-0.26	17,812	0.92+-0.19
Roary 90%	30,720,262	1.00+-0.00	2,993	1.00+-0.01	231,592,623	0.96+-0.20	46,319	0.95+-0.16
Roary 50%	36,550,488	1.00+-0.02	1,712	1.00+-0.01	323,115,520	0.93+-0.26	26,071	0.92+-0.20
PanX	43,018,578	1.00+-0.03	1,150	1.00+-0.01	392,821,552	0.92+-0.28	18,227	0.94+-0.16

	SCC	MCC	orphans	non-orphans clusters
SynerClust without inparalogs	1,329	0	28,387	40,827
SynerClust	1,156	233	19,315	33,622
Hieranoid2	1,037	236	76,542	28,131
Roary 90%	172	0	29,738	61,923
Roary 50%	1,062	2	15,791	36,933
PanX	806	635	12,548	26,864

Table S3: b) *Enterobacteriaceae*

Table S3: Table of all scores for each method, with count of number of pairs and clusters scored in each case. Considering an equal score, a higher number of pairs or a lower number of clusters scored indicates more complete clusters. Standard deviation values are indicated.
